# Granulocyte colony-stimulating factor promotes regeneration of severed facial nerve in rats

**DOI:** 10.3389/fnins.2024.1442614

**Published:** 2024-12-05

**Authors:** Yoko Fujimaki, Kenji Kondo, Hironobu Nishijima, Shu Kikuta, Tatsuya Yamasoba

**Affiliations:** ^1^Department of Otorhinolaryngology and Head and Neck Surgery, Graduate School of Medicine, The University of Tokyo, Tokyo, Japan; ^2^Department of Otolaryngology-Head and Neck Surgery, Nihon University School of Medicine, Tokyo, Japan

**Keywords:** granulocyte colony-stimulating factor (G-CSF), granulocyte colony-stimulating factor receptor (G-CSFR), facial nerve, regeneration, drug delivery system, electroneurography (ENoG)

## Abstract

**Background and aim:**

The administration of growth and neurotrophic factors has been attempted experimentally as a new therapeutic strategy for severe facial paralysis. Granulocyte colony-stimulating factor (G-CSF) has an effect on the treatment of central nervous system injuries, such as cerebral infarction and spinal cord injury. This study aimed at examining the effects of G-CSF on facial nerve regeneration in rats.

**Methods:**

The left facial nerve of rats was either partially resected (resection group) or severed and sutured (suture group) at the main trunk outside the temporal bone. In each surgical group, saline or G-CSF was administered via the gelatin hydrogel drug delivery system. The suture group was further divided into two subgroups for the late administration of G-CSF (2 weeks after surgical treatment) or immediate administration of G-CSF after surgical treatment. Recovery of the facial nerve was assessed by the evaluation of facial movements (after 12 weeks), complex muscle action potential amplitude measurements (after 2, 4, 8, and 12 weeks), electroneurography values (after 12 weeks), and histological evaluation (comparison of myelinated axon diameters among the groups).

**Results:**

Recovery of the function and morphology of damaged nerves was faster in the suture groups than in the resection group. In the suture groups, recovery was faster for G-CSF-treated rats than for saline-treated rats. Furthermore, recovery was faster in the group that received G-CSF immediately after surgical treatment than in the group that received G-CSF 2 weeks later. However, the group that received G-CSF 2 weeks later also showed faster recovery than did the control group.

**Conclusion:**

G-CSF effectively promoted nerve regeneration during facial nerve paralysis. Thus, G-CSF may be a potential treatment strategy for injured facial nerves as it has been safely administered in clinical treatments for hematological diseases.

## 1 Introduction

Facial nerve palsy is a common disease with varying degree of neurological impairment from mild to complete. Facial paralysis is not a fatal disease but has a significant impact on the mental and social life of the patient owing to esthetic challenges. Recovery from paralytic symptoms can take several months. In cases of severe facial nerve degeneration in particular, complete recovery has not yet been achieved. This has a significant impact on the quality of life of the patients.

Currently, the pharmaceutical treatments for peripheral facial nerve paralysis include steroids, antivirals, vitamins, circulatory agents, and vasodilators. Although steroids have been established as effective medications for the prevention of facial nerve degeneration, no drug has been proven effective in promoting the regeneration of damaged facial nerves. Growth factors and neurotrophic factors have been tested as novel treatment strategies for damaged facial nerves; however, they are still not approved for clinical application (Sendtner et al., 1991; Hughes et al., 1993; Spector et al., 1993; Yan et al., 1995; Koyama et al., 2003; Hato et al., 2012).

Granulocyte colony-stimulating factor (G-CSF), a glycoprotein with a molecular weight of approximately 19 KDa and 174 amino acids, belongs to the cytokine family and regulates granulocyte production (Nicola et al., 1983; Nagata et al., 1986; Fukunaga et al., 1990; Demetri and Griffin, 1991; Lieschke et al., 1994). Small amounts of G-CSF are homeostatically produced by monocytes, macrophages, vascular endothelial cells, fibroblasts, and bone marrow-derived mesenchymal cells. Because G-CSF increases the number of leukocytes and mobilizes hematopoietic stem cells to the peripheral blood, it has been used in the treatment of leukopenia in hematologic diseases and pancytopenia during chemotherapy (Ohno et al., 1990; Usuki et al., 2002; Aapro et al., 2011; Potosky et al., 2011). Recent studies have reported that G-CSF also has central neuroprotective effects and is a potential drug for cerebral infarction (Schabitz et al., 2003; Park et al., 2005; Schneider et al., 2005a; Schneider et al., 2005b; Solaroglu et al., 2006a; Solaroglu et al., 2006b; Solaroglu et al., 2007; Rahi et al., 2021), spinal cord injury (Koda et al., 2007; Nishio et al., 2007; Kawabe et al., 2011; Aschauer-Wallner et al., 2021), and other neurological diseases (Meuer et al., 2006; Tanaka et al., 2006; Laske et al., 2009; Sanchez-Ramos et al., 2009; Vafaei Mastanabad et al., 2023). Although the effects of G-CSF on the central nervous system have been reported, only a few studies have investigated its application in peripheral nerve therapy.

G-CSF may be a good candidate for the treatment of facial nerve paralysis if it is effective in promoting facial nerve regeneration as it has been safely used in clinical settings for hematologic diseases. Thus, this study aimed at examining the potential of G-CSF for the treatment of facial nerve paralysis using a rat model. We investigated its effects on differential facial nerve damage and the optimal administration time to restore facial nerve paralysis.

## 2 Materials and methods

### 2.1 Experimental animals

Sprague Dawley (SD) male rats aged 10–12 weeks (CLEA Japan, Inc.) were used in this study. The rats were reared under free feeding and drinking conditions during the light period from 9:00 to 21:00 with a room temperature of 23°C. All animal experiments were conducted in accordance with the University of Tokyo’s Regulations for the Conduct of Animal Experiments and the NIH Guide for the Care and Use of Laboratory Animals. Efforts were made to minimize the suffering of laboratory animals.

### 2.2 Preparation of drugs

G-CSF has a short half-life; therefore, it must be administered daily. To overcome this challenge, a drug delivery system with an *in vivo* absorbable gelatin hydrogel was used (MedGel^®^, MedGEL CO., Ltd, Kyoto, Japan).

A bioabsorbable gelatin hydrogel is a water-insoluble gelatin hydrogel formed by crosslinking gelatin. G-CSF is retained by electrostatic interactions in the gelatin hydrogel. In the *in vivo* implanted gelatin hydrogel, G-CSF is slowly released over a period of approximately 2 weeks as gelatin is degraded by collagenases and other degradative enzymes secreted by the tissue cells (Tabata, 2003; Komobuchi et al., 2010).

In this experiment, lenograstim (Neutrogin Injection, 250 μg; Chugai Pharmaceutical Co., Ltd., Tokyo, Japan) was used for the G-CSF preparation. Powdered lenograstim (250 μg) was dissolved in 350 μL of sterile water (hereinafter referred to as G-CSF solution). The G-CSF solution [150 μg/kg body weight (BW); 210 μL of G-CSF solution/kg BW] was added to gelatin hydrogel (as 10 μL of G-CSF solution per 1 mg of gelatin hydrogel) (Kawamura et al., 2011; Nakaguchi et al., 2012; Simoes and de Oliveira, 2012). The gelatin hydrogels were left at 37°C for 1 h to allow the G-CSF solution to fully impregnate the gelatin hydrogels. In the control group, saline (210 μL/kg BW) was added to the gelatin hydrogel instead of the G-CSF solution, and the gelatin hydrogel was left at 37°C for 1 h to become fully impregnated.

In this experiment, the drug (G-CSF or saline) impregnated into the gelatin hydrogel was subcutaneously administered into the back of each rat. A 5-mm incision was placed on the mid-back at the level of the lower end of the shoulder blades, the hydrogel was subcutaneously embedded, and the incision was sutured and closed.

### 2.3 Surgical treatment

The facial nerve was treated at the extratemporal main trunk. The SD rats were anesthetized with an intraperitoneal injection of ketamine at 40 mg/kg BW (Daiichi Sankyo Healthcare Co., Ltd., Tokyo, Japan) and 10 mg/kg BW xylazine (Elanco Japan Co., Ltd., Tokyo, Japan), followed by inhalation maintenance anesthesia with isoflurane (Pfizer Japan Inc., Tokyo, Japan). The skin anterior to the left ear was incised to expose the parotid gland and facial nerve over the masseter muscle. The parotid gland was detached from the masseter and facial nerves; the main trunk of the facial nerve was exposed and severed using scissors. In the resection model, the peripheral part of the facial nerve was resected 10 mm from the cutting point to maintain the distance between the severed nerves (Tews et al., 1994). In the severed-and-sutured model, the epineural membrane of the left facial nerve was sutured end-to-end using a micro-needle (9–0 nylon monofilament; Keisei Medical Industries, Tokyo, Japan) immediately after transection. The neurotomy or severed-and-sutured site was covered by the parotid gland. The skin was sutured using a non-absorbable suture (5–0 nylon monofilament; Akiyama Medical MFG Co., Ltd., Tokyo, Japan).

### 2.4 Experimental protocol

**Figure 1:**
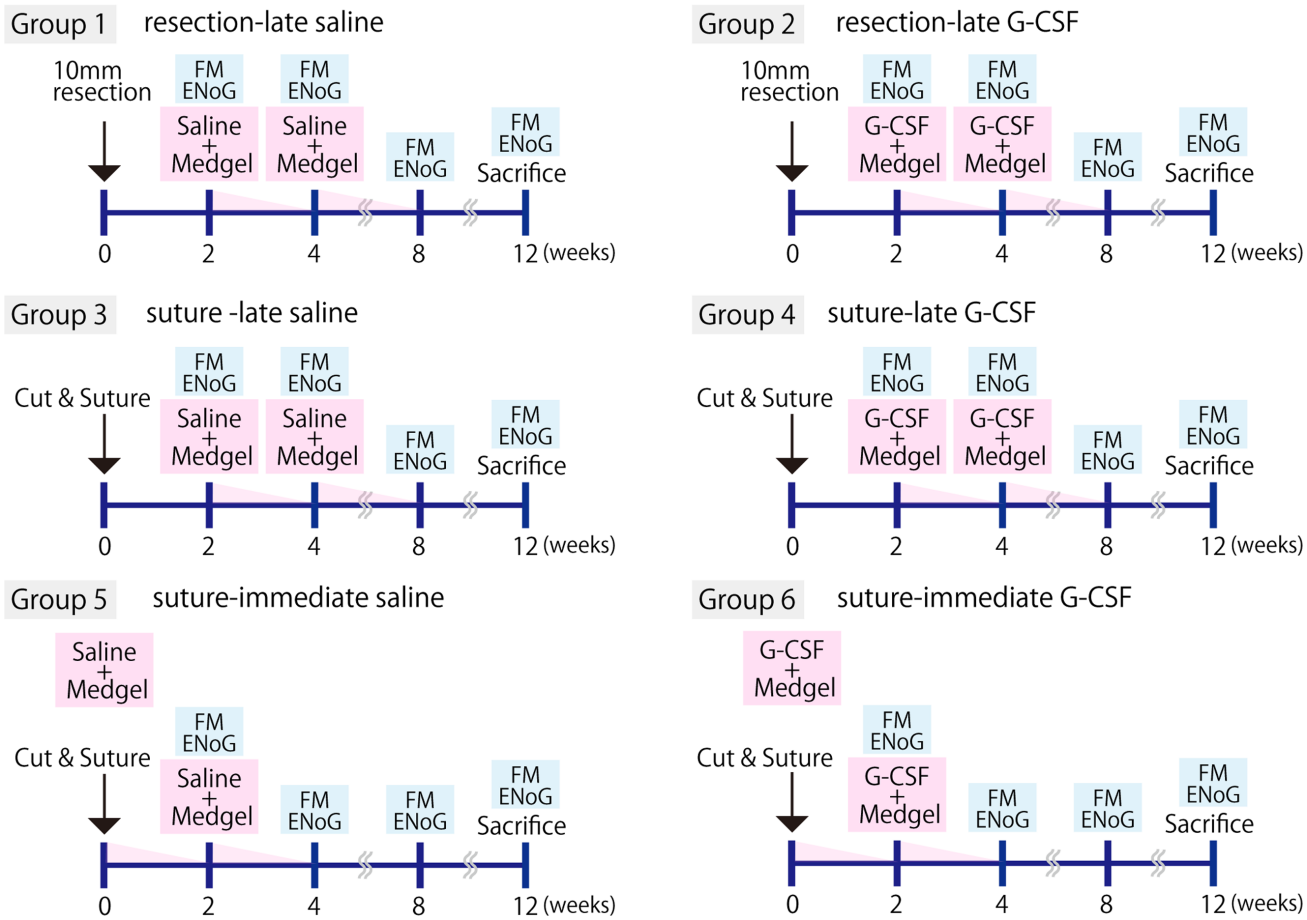
Experimental protocols. The experiment was performed with six groups (five animals per group). Facial nerve resection (resection group) or severance followed by surgical suturing (suture group) was performed. Either G-CSF 150 μg/kg (210 μL/kg of the solution) or an equivalent amount of saline was administered immediately after surgery (−immediate group) or 2 weeks later (−late group). In all cases, the drug was percutaneously administered into the back by inserting Medgel soaked in the G-CSF solution or saline. This procedure was repeated twice. Evaluation of facial movements (FM) and electroneurography (ENoG) was performed at 2, 4, 8, and 12 weeks, and tissue samples were harvested at 12 weeks.

The partial facial nerve resection or severed-and-sutured model was assigned to six groups (five animals in each group; Figure 1).

Group 1, the left facial nerve was partially resected and the saline solution was administered 2 and 4 weeks later (hereafter, “resection-late saline”).

Group 2, the left facial nerve was partially resected and G-CSF was administered 2 and 4 weeks later (hereafter, “resection-late G-CSF”).

Group 3, the left facial nerve was severed and sutured, and saline solution was administered 2 and 4 weeks later (hereafter, “suture-late saline”).

Group 4, the left facial nerve was severed and sutured, and G-CSF was administered 2 and 4 weeks later (hereafter, “suture-late G-CSF”).

Group 5, the left facial nerve was severed and sutured, and saline solution was administered immediately and 2 weeks later (hereafter, “suture-immediate saline”).

Group 6, the left facial nerve was severed and sutured, and G-CSF was administered immediately and 2 weeks later (hereafter, “suture-immediate G-CSF”).

In the pilot study, the group that underwent skin incision and wound closure procedures in the same manner was designated as the sham operation group. No facial nerve palsy or effects on facial muscles were observed in this group, confirming that the surgical procedure itself did not cause any adverse effects. To avoid complications, the sham surgery group was excluded from the present comparative study.

### 2.5 Evaluation of facial movements

**Figure 2:**
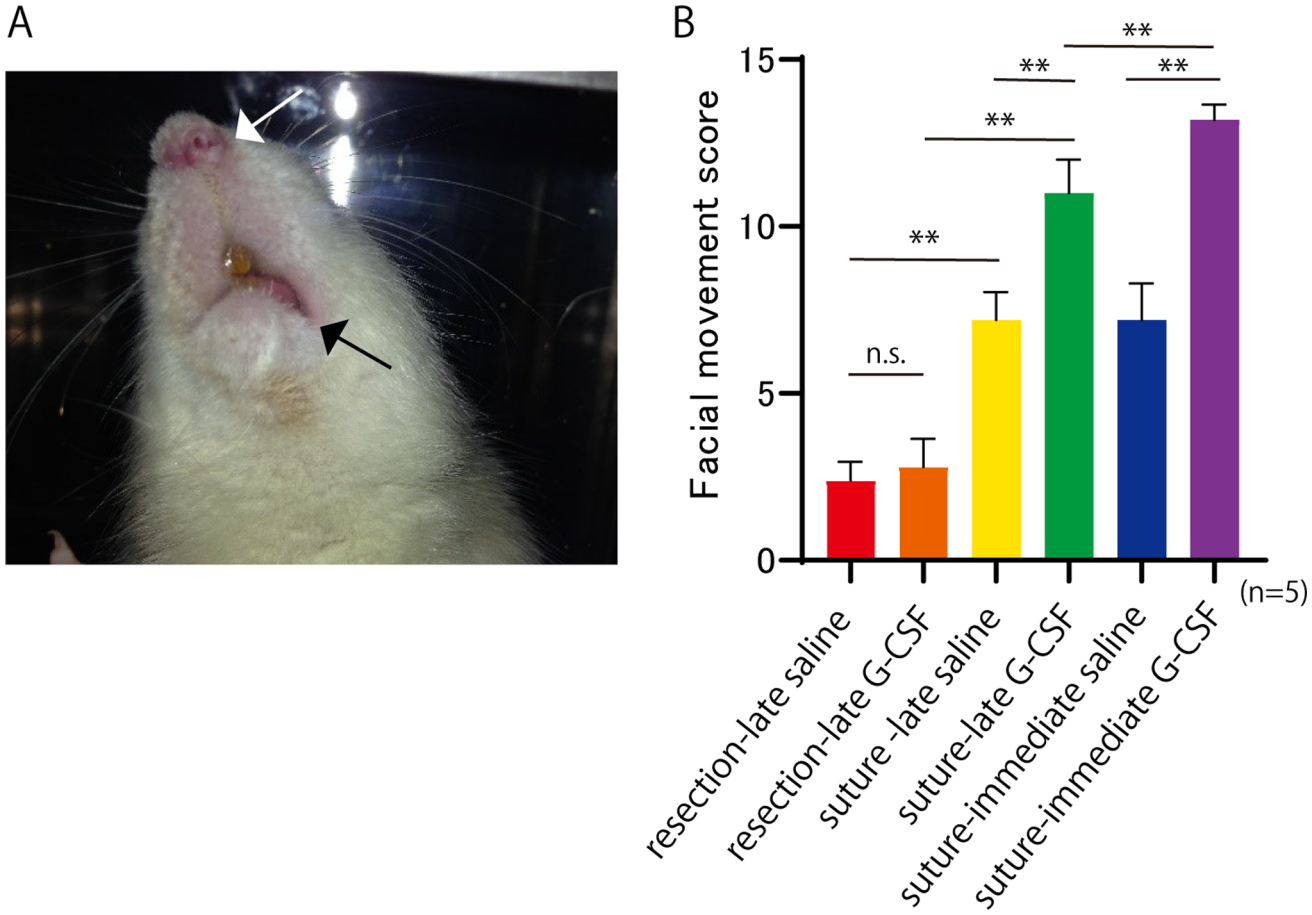
Facial paralysis at 12 weeks after facial nerve resection. **(A)** Asymmetry of the face, including the shape of the nasal wings (white arrow) and corners of the mouth (black arrow), indicates facial paralysis. **(B)** Facial movement score at 12 weeks after the surgical treatment. The score for normal facial movement is 15. Significant differences are observed among groups (*n* = 5 in each group). *p < 0.05, **p < 0.01, ns = no significant difference.

Facial movements were recorded at 2, 4, 8, and 12 weeks after the surgical treatment. The facial movements were scored and evaluated in a blinded manner. The following five items were evaluated. Symmetry at rest, and movement of four facial areas: the eyelids, alae of the nose, corners of the mouth, whiskers. The evaluation criteria for symmetry at rest were as follows: marked asymmetry (0 points), relatively clear asymmetry (1 point), slight asymmetry (2 points), and symmetry (3 points). The evaluation criteria for movement were as follows: no movement at all (0 points), slight movement (1 point), obvious movement but with a left-right difference (2 points), and no difference between the left and right sides (3 points). The score for normal facial movement was 15. The mean total values were compared between groups. A representative case involving a rat with complete facial nerve paralysis is shown in Figure 2A. The face was asymmetrical, with paralysis of the left angle of the mouth.

### 2.6 Evaluation of electroneurography findings

Electroneurography (ENoG) is an electrophysiological diagnostic method for evaluating peripheral facial nerve palsy and is an objective measure of complex muscle action potentials (CMAPs). Electrical stimulation is applied to the facial nerve from the stylomastoid foramen, and primarily, contractions in the orbicularis oris muscle are recorded using electrodes. The ratio of CMAP amplitude on the lesion side to that on the normal side (ENoG value) is used to evaluate the prognosis of peripheral facial nerve palsy (Gantz et al., 1984). CMAP amplitudes in healthy human subjects show an average variation of 25% between the left and right sides of the face (Gavilán et al., 1985).

ENoG was performed 2, 4, 8, and 12 weeks after surgical treatment (Figure 1) using the electromyographic device (Neuropack MEB-2000; Nihon Kohden Corporation, Tokyo, Japan). The anterior part of the left ear was opened, and a stimulating electrode needle (Unique Medical Co., Ltd., Tokyo, Japan) was applied as a negative electrode on the central side of the part that had been partially resected or severed and sutured, near the foramen magnum, and a positive electrode on the posterior skin of the left ear. The recording was performed on the orbicularis oris muscle. The negative recording electrode was placed 2 mm to the left of the midline of the upper lip, and the positive recording electrode was placed below the midline of the lower lip. The stimulus intensity was uniformly set at 2 mA for the maximum stimulus test (MST), and pulse duration was 0.2 ms. The CMAP amplitude was measured five times per rat for each group of five rats. The average CMAP amplitude values for each group were determined at 2, 4, 8, and 12 weeks after the surgical treatment.

The ENoG values were calculated as the percentage of the amplitude value of the treated side relative to the non-treated side using the mean amplitude values of the left and right CMAPs for each animal after 12 weeks of surgical treatment. The mean ENoG values were calculated for each group.

### 2.7 Tissue fixation and preparation

Each rat was deeply anesthetized with 100 mg/kg ketamine hydrochloride and 10 mg/kg xylazine hydrochloride and perfusion-fixed with 10% neutral-buffered formalin 12 weeks after surgical treatment.

After decapitation, the facial tissues were soaked in 10% neutral-buffered formalin for 14 days. The tissues were then immersed in 10% formic acid solution for 5 days for decalcification. The tissue was then embedded in paraffin and cut at a 4-μm thickness in a coronal plane.

### 2.8 Histological evaluation

To detect myelin sheaths, which are indicators of nerve activity, Klüver–Barrera (KB) staining was performed on sections of the facial nerve distal to the cutting point (buccal branch) 12 weeks after surgical treatment. The diameters of 10 myelinated axons from each rat were measured using ImageJ software (National Institutes of Health, United States).^1^ The mean values calculated for each group were compared. Additionally, recovery from muscle atrophy in the orbicularis oculi and orbicularis oris muscles was assessed by hematoxylin and eosin (H&E) staining 12 weeks post-surgery for each group. Orbicularis oculi muscle samples were obtained from a vertical section at the midline of the upper eyelid of the rat, while orbicularis oris muscle samples were obtained from a coronal section of the upper lip centered at the philtrum.

### 2.9 G-CSF receptor expression in facial nerve nuclei and peripheral nerves

To evaluate the G-CSF receptor (G-CSFR) expression in the facial nerve and facial nerve nucleus after partial resection of the facial nerve, the main trunk of the left facial nerve of the SD rats was partially resected as described. G-CSFR expression in the facial nerve nucleus was assessed on postoperative day 5, and that in a peripheral nerve (buccal branch) was assessed on postoperative week 2.The rats were fixed with 10% neutral-buffered formalin for 5 days, and the proximal facial nerve and brainstem were harvested. The paraffin section of the brainstem was cut at 4-μm thickness in a horizontal plane. Sections of the facial nerve nucleus were selected based on a rat brain atlas (Paxinos and Watson, 2013). The sections were deparaffinized and rehydrated in a xylene-ethanol series, followed by antigen activation by microwaving the section at 95°C for 10 min in 10 mM citrate buffer (Agilent Technologies, California, United States). They were then immersed in a 3% hydrogen peroxide solution for 10 min to block endogenous peroxidases. After treatment with the blocking solution (Blocking One^®^; NAKALAI TESQUE, INC, Kyoto, Japan) for 30 min at room temperature to reduce nonspecific antibody reaction, anti-G-CSFR antibody (diluted at 1:300 in an antibody diluent, Bioss Antibodies Inc., Massachusetts, United States) was added as a primary antibody and allowed to react overnight at 4°C. After washing with PBS, the secondary antibody (Molecular probes, Alexa Fluor^®^ 568 goat anti-rabbit IgG, Abcam, plc, Cambridge, United Kingdom) was diluted 200x with antibody diluent (NAKALAI TESQUE, Inc.), added to the sections, and incubated at 37°C for 30 min in the dark box. The sections were then mounted with the antifading medium (Vectashield^®^; VECTOR LABORATERIES, Inc., CA, United States) and observed using a KEYENCE BZ-9000 microscope.

### 2.10 Statistical methods

The experimental results are expressed as mean ± standard deviation. One-way analysis of variance (ANOVA) followed by Bonferroni’s multiple comparisons test or Dunnett’s multiple comparisons test was performed using GraphPad Prism version 10.2.0 for Windows (GraphPad Software, Boston, MA, United States).^2^ A *p*-value of <0.05 was considered statistically significant.

## 3 Results

### 3.1 Evaluation of facial movements

Representative images of the rats with complete facial nerve paralysis are shown in Figure 2A. Facial movements were scored based on the five previously described items (five rats per group), and the mean values are shown in Figure 2B. One-way ANOVA showed a significant difference among the six groups [*F*(5, 24) = 135.8; *p* < 0.01].

In the comparison of the two surgical procedures, the mean score was significantly higher in the suture-late saline group than in the resection-late saline group (Bonferroni’s multiple comparison test; *p* < 0.01). The mean score was also significantly higher in the suture-late G-CSF group than in the resection-late G-CSF group (Bonferroni’s multiple comparison test; *p* < 0.01).

Regarding the comparison between the control and G-CSF groups, the mean score was significantly higher in the suture-late G-CSF group than in the suture-late saline group (Bonferroni’s multiple comparison test; *p* < 0.01). The mean score was also significantly higher in the suture-immediate G-CSF group than in the suture-immediate saline group (Bonferroni’s multiple comparison test, *p* < 0.01). There was no significant difference in facial movement between the two partial resection groups, namely the resection-late saline and resection-late G-CSF groups (Bonferroni’s multiple comparison test; *p* > 0.9999).

In comparisons between groups with G-CSF treatments at different postsurgical timings, the mean score was significantly higher in the suture-immediate G-CSF group than in the suture-late G-CSF group (Bonferroni’s multiple comparison test; *p* < 0.01).

### 3.2 CMAP amplitude

**Figure 3:**
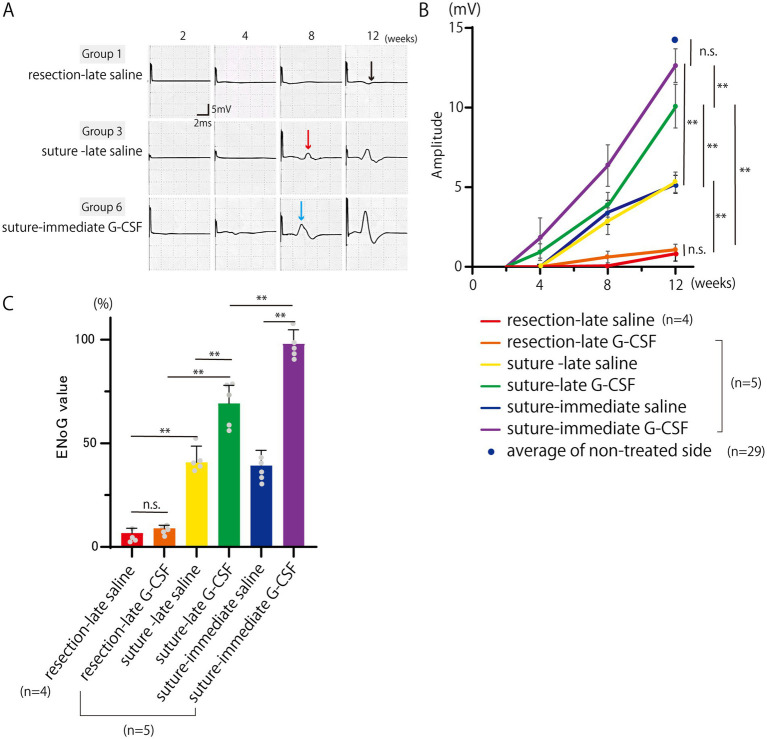
Electroneurography (ENoG) assessments of the facial nerve. **(A)** Representative CMAP waveforms of the orbicularis oris muscle. In the resection-late saline group, a slight baseline oscillation is observed even after 12 weeks (black arrow), whereas in the suture-late saline (red arrow) and suture-immediate G-CSF groups (blue arrow), a clear CMAP waveform is observed after 8 weeks. The CMAP waveforms are larger in the suture-immediate G-CSF group than in the suture-late saline group. **(B)** Temporal changes in the CMAP amplitude. The CMAP amplitude is measured in each group (*n* = 5 in all groups except the resection-late saline group, where one animal died during ENoG measurement [*n* = 4]). There are significant differences between groups at 12 weeks after the surgical treatment. In each group, a comparison with the average CMAP amplitude on the non-treated side in the six groups at week 12 (*n* = 29) was also performed. There was no significant difference between the mean CMAP amplitude on the treated side in the suture-immediate G-CSF group and that on the non-treated side in the six groups. **p < 0.01. ns = no significant difference. **(C)** Comparison of ENoG values at 12 weeks after the surgical treatment. Significant differences are observed among groups (*n* = 5 in all groups except the resection-late saline group [*n* = 4]). *p < 0.05, **p < 0.01, ns = no significant difference.

The representative CMAP waveforms are shown in Figure 3A. In the resection-late-saline group, only a slight baseline oscillation was observed even after 12 weeks, whereas in the suture-late-saline and suture-immediate G-CSF groups, a clear CMAP waveform was observed after 8 weeks. The CMAP waveforms were larger in the suture-immediate G-CSF group than in the suture-late saline group.

The CMAP amplitude measurements are shown in Figure 3B. All groups were unresponsive to electrical stimulation immediately after surgical treatment; however, in the suture-late G-CSF and suture-immediate G-CSF groups, CMAP reappeared 4 weeks after surgical treatment.

Statistical analysis was performed for the mean CMAP amplitudes at 12 weeks [*n* = 5 in all groups except the resection-late saline group (*n* = 4), where one animal died during evoked electromyography measurement]. One-way ANOVA showed significant differences among the six groups on the treated side [*F*(5, 23) = 141.3; *p* < 0.01], with no significant differences among the six groups on the non-treated side [*F*(5, 23) = 0.83; *p* = 0.53].

In the comparison of the two surgical procedures, the mean amplitude was significantly higher in the suture-late saline group than in the resection-late saline group (Bonferroni’s multiple comparison test; *p* < 0.01). The mean amplitude value was also significantly higher in the suture-late G-CSF group than in the resection-late G-CSF group (Bonferroni’s multiple comparison test; *p* < 0.01).

In the comparison between the control and G-CSF groups, the mean CMAP amplitude was significantly higher in the suture-late G-CSF group than in the suture-late saline group (Bonferroni’s multiple comparison test; *p* < 0.01). The mean CMAP amplitude was also significantly higher in the suture-immediate G-CSF group than in the suture-immediate saline group (Bonferroni’s multiple comparison test; *p* < 0.01). There was no significant difference in the mean CMAP amplitude between the two partial resection groups, namely the resection-late saline and resection-late G-CSF groups (Bonferroni’s multiple comparison test; *p* > 0.9999).

In comparisons between groups with G-CSF administration at different timings, the mean CMAP amplitude was significantly higher in the suture-immediate G-CSF group than in the suture-late G-CSF group (Bonferroni’s multiple comparison test; *p* < 0.01).

The average CMAP amplitudes at week 12 on the non-treated sides in the six groups (*n* = 29) are also plotted in Figure 3B. There was no significant difference between the mean CMAP amplitude on the treated side in the suture-immediate G-CSF group and that on the non-treated side in the six groups (Dunnett’s multiple comparison test; *P* = 0.6911). In the other groups, the mean values on the treated sides were significantly lower than the mean value on the non-treated sides in the six groups (Dunnett’s multiple comparison test; *p* < 0.01).

### 3.3 ENoG values 12 weeks after the surgical treatment

The comparison between the ENoG values 12 weeks after the surgical treatment is shown in Figure 3C [*n* = 5 in all groups except the resection-late saline group (*n* = 4)]. One-way ANOVA showed significant differences among the six groups [*F*(5, 23) = 141.0, *p* < 0.01].

In the comparison between the two surgical procedures, the mean ENoG value was significantly higher in the suture-late saline group than in the resection-late saline group (Bonferroni’s multiple comparison test; *p* < 0.01). The mean ENoG value was also significantly higher in the suture-late G-CSF group than in the resection-late G-CSF group (Bonferroni’s multiple comparison test; *p* < 0.01).

In the comparison between the control and G-CSF groups, the mean ENoG values were significantly higher in the suture-late G-CSF group than in the suture-late saline group (Bonferroni’s multiple comparison test; *p* < 0.01); they were also significantly higher in the suture-immediate G-CSF group than in the suture-immediate saline group (Bonferroni’s multiple comparison test; *p* < 0.01). There was no significant difference in the mean ENoG values between the two partial resection groups, namely the resection-late saline and resection-late G-CSF groups (Bonferroni’s multiple comparison; *p* > 0.9999).

In comparisons of groups with G-CSF treatments at different postoperative timings, the mean ENoG value was significantly higher in the suture-immediate G-CSF group than in the suture-late G-CSF group (Bonferroni’s multiple comparison test; *p* < 0.01).

### 3.4 Histological evaluation of peripheral facial nerve

**Figure 4:**
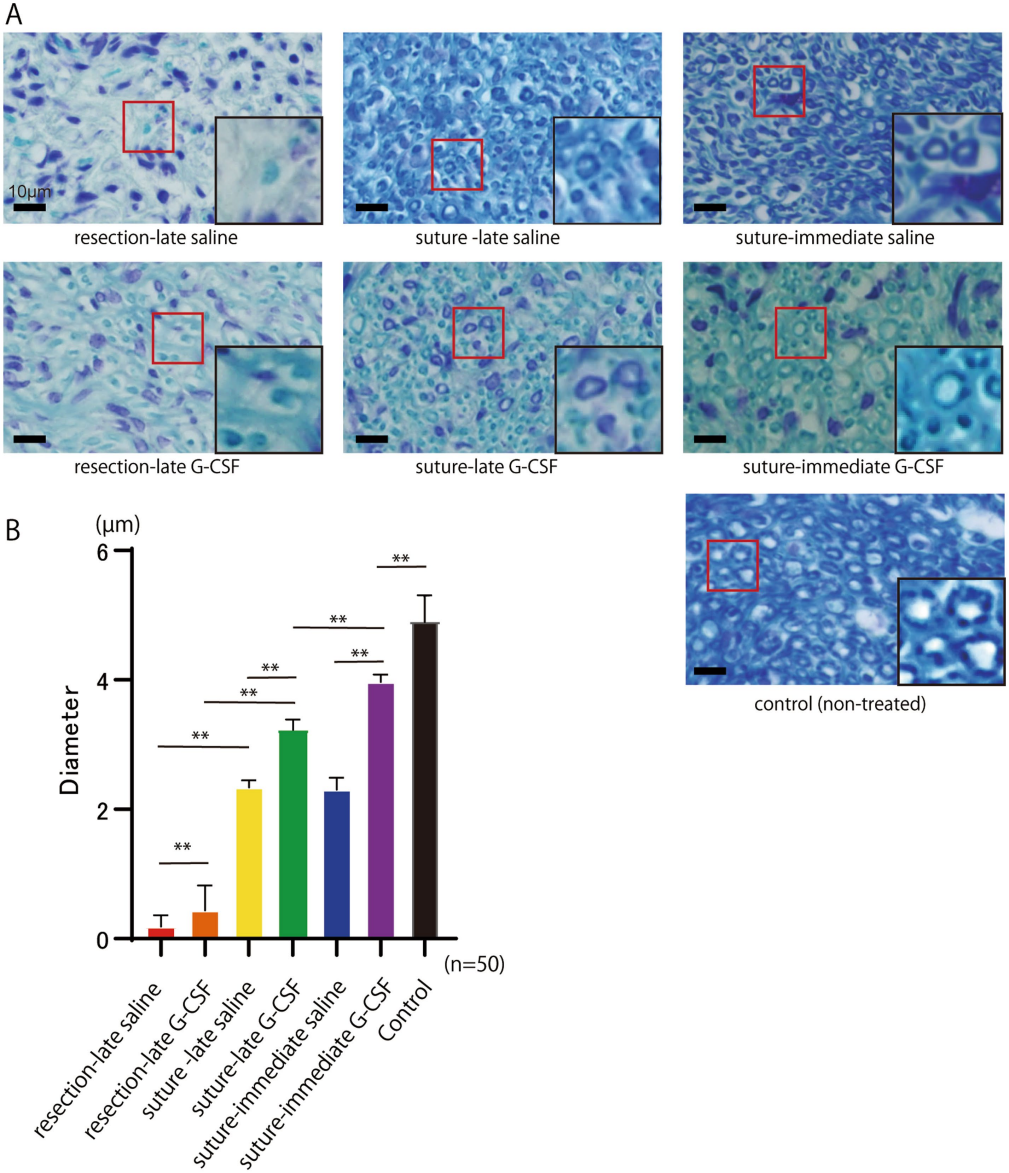
Histological evaluation of the peripheral facial nerve (buccal branch) assessed approximately 18 mm from the cutting point 12 weeks after the surgical treatment. **(A)** Abundant neomyelination is observed in the suture groups compared to the resection groups. The red squares indicate new myelin sheaths. Scale bar =10 μm. The squares on the lower right show enlarged views of the areas within the red squares on the peripheral facial nerve (buccal branch). The diameters of the myelinated axons in the suture groups look larger than those in the resection groups. The diameters of the axons in the suture G-CSF groups look larger than those in the suture-saline groups. **(B)** The mean diameter of myelinated axons in the buccal branch at 12 weeks after the surgical treatment. Significant differences are observed among groups (*n* = 50 in each group). The mean axonal diameter in the suture-immediate G-CSF group is still significantly different from that in the control group; however, it is closest to that in the control group among all six groups. *p < 0.05, **p < 0.01, ns no significant difference.

The histology of KB staining of the peripheral facial nerve was assessed approximately 18 mm from the cutting point 12 weeks after the operation (Figures 4A). The mean diameter of the myelinated axons (10 axons per nerve; *n* = 50 per group) is shown in Figure 4B. One-way ANOVA showed significant differences among the six groups [*F*(5,294) = 2537; *p* < 0.01].

In the comparison between the two different surgical procedures, the mean axonal diameter was significantly larger in the suture-late saline group than in the resection-late saline group (Bonferroni’s multiple comparison test; *p* < 0.01); it was also significantly larger in the suture-late G-CSF group than in the resection-late G-CSF group (Bonferroni’s multiple comparison test; *p* < 0.01).

Regarding the comparison between the control and G-CSF groups, the mean axon diameter was significantly larger in the suture-late G-CSF group than in the suture-late saline group (Bonferroni’s multiple comparison test; *p* < 0.01). Moreover, it was significantly larger in the suture-immediate G-CSF group than in the suture-immediate saline group (Bonferroni’s multiple comparison test; *p* < 0.01). The mean axonal diameter was significantly larger in the resection-late G-CSF group than in the resection-late saline group (Bonferroni’s multiple comparison test; *p* < 0.01).

In comparisons between groups treated with G-CSF at different postoperative timings, the mean axon diameter was significantly larger in the suture-immediate G-CSF group than in the suture-late G-CSF (Bonferroni’s multiple comparison test; *p* < 0.01).

The mean axonal diameter in the same area of the buccal branch in the control group without facial nerve amputation (*n* = 50) was 4.92 ± 0.38 μm. The mean axonal diameter in the suture-immediate G-CSF group was 3.96 ± 0.11 μm. There was still a significant difference in the mean axonal diameter between the two groups (Dunnett’s multiple comparison test; *p* < 0.01); however, the mean axonal diameter in the suture-immediate G-CSF group was closest to that in the control group among all six groups.

### 3.5 Facial muscle atrophy

**Figure 5:**
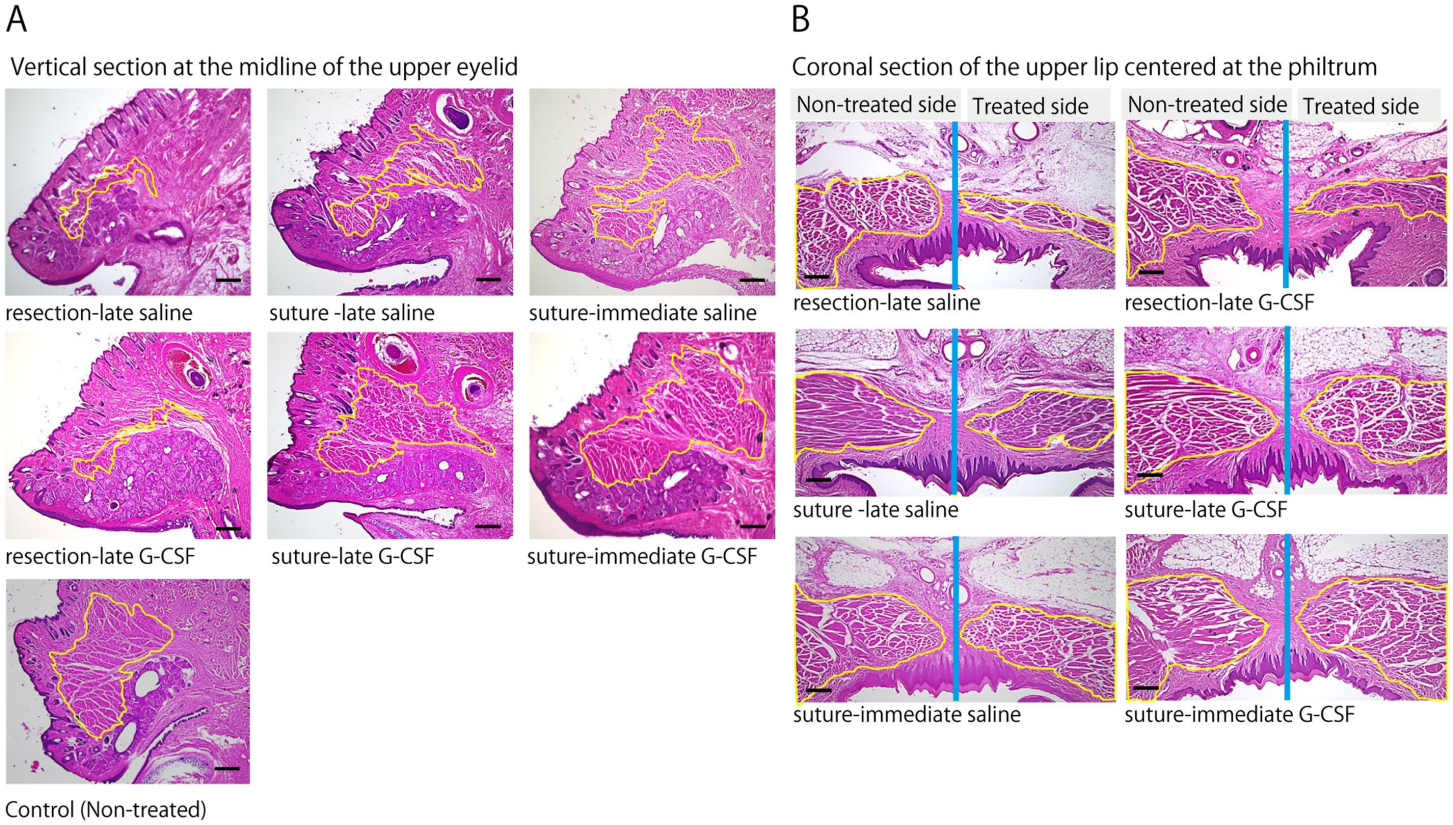
Pathological comparison of muscles innervated by the facial nerve. **(A)** Representative images of the orbicularis oculi muscles on the treated sides and the non-treated control 12 weeks after the surgical treatment of facial nerve (H&E staining); samples were obtained from a vertical section at the midline of the upper eyelid. Compared to the non-treated control, the orbicularis oculi muscles (yellow-circled area) show marked atrophy in the resection groups. In contrast, the muscle volume in the suture- G-CSF groups appears to have recovered close to the level of that in the non-treated control. Scale bar =300 μm. **(B)** Representative images of the orbicularis oris muscles on the treated and non-treated sides 12 weeks after the surgical treatment of facial nerve (H&E staining); samples were obtained from a coronal section of the upper lip centered at the philtrum. The yellow-circled area indicates the orbicularis oris muscle, and the blue line indicates the midline of the head. Significant atrophy of the orbicularis oris muscle is observed on the treated side in the resection groups. In the suture groups, muscle atrophy is less pronounced compared to that in the resection groups. In the suture-G-CSF groups, muscle atrophy shows recovery to a size comparable to that in the non-treated side. Scale bar =300 μm.

The degree of recovery from muscle atrophy after 12 weeks in each group was examined using H&E staining of the orbicularis oculi (upper eyelid) and orbicularis oris muscles (Figure 5).

Representative images of the orbicularis oculi muscle (upper eyelid) are shown in Figure 5A. Compared to the control group, significant atrophy of the orbicularis oculi muscle was observed at 12 weeks in the resection groups (resection-late saline and resection-late G-CSF groups). In contrast, muscle atrophy was less severe in the suture groups (suture-late saline, suture-late G-CSF, suture-immediate saline, and suture-immediate G-CSF groups). Furthermore, in the suture and G-CSF-administered groups, the muscle volume recovered to a level comparable to that of the control group. Similarly, H&E staining of the orbicularis oris muscle showed marked atrophy in the resection group (Figure 5B). However, muscle atrophy was less pronounced in the suture groups compared to the resection groups, with recovery in the suture and G-CSF-administered group to a level similar to that of the non-treated side.

### 3.6 G-CSFR expression in facial nerve nuclei and facial nerve

**Figure 6:**
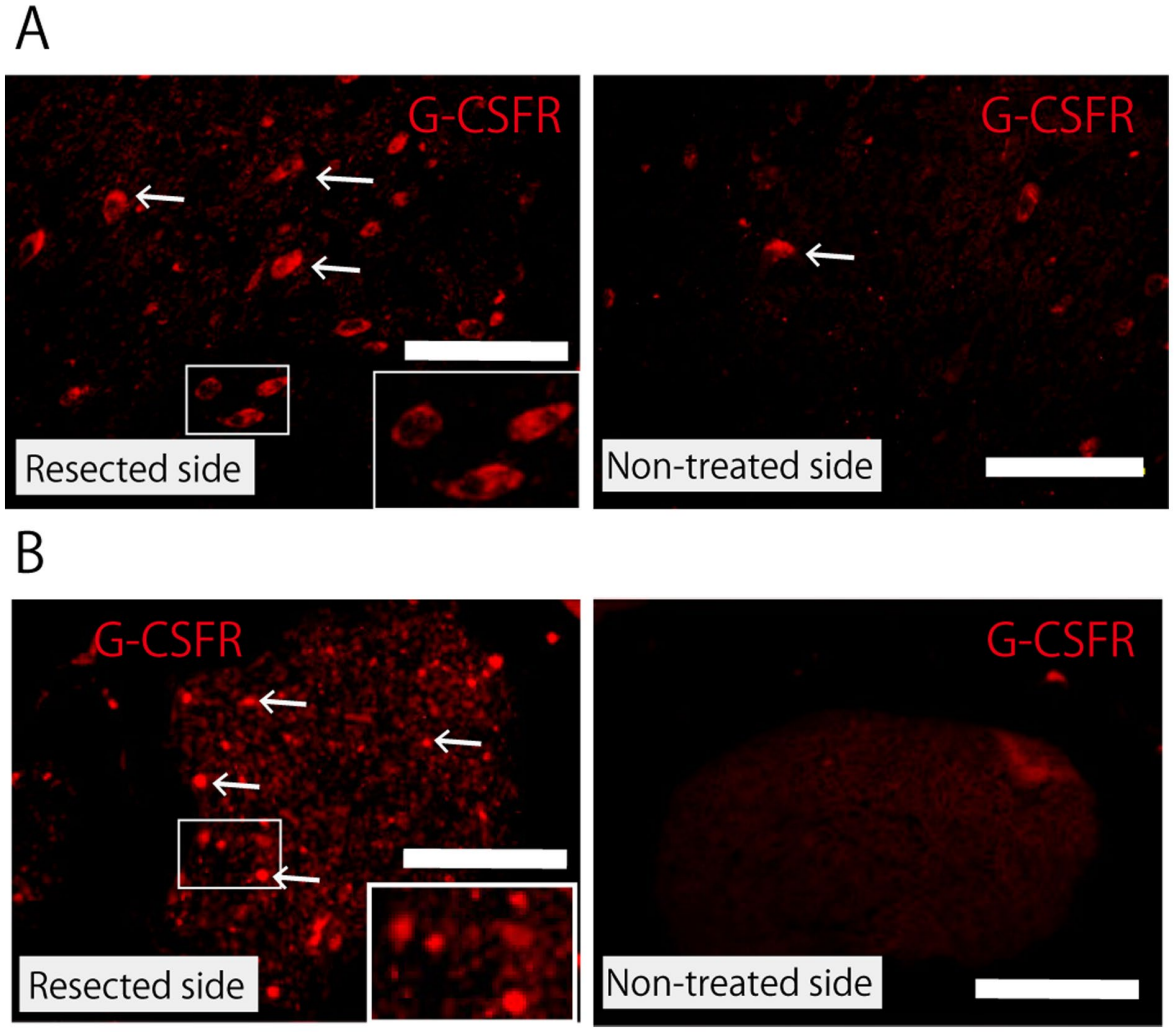
Expression of G-CSFR. **(A)** Facial nerve nucleus 5 days after partial facial nerve resection immunostained with anti-G-CSFR antibody. Compared with the non-treated side, the facial nerve nucleus on the resected side shows upregulation in the expression of G-CSFR. The arrow indicates G-CSFR-positive cells. Scale bar =100 μm. The rectangle in the lower right corner of the figure for the resected side shows a magnified view of the area within the small rectangle. **(B)** Peripheral facial nerve (buccal branch) 2 weeks after partial nerve resection immunostained with anti-G-CSFR antibody. Immunoreactivity for G-CSFR is observed in the peripheral nerve on the resected side, but not on the non-treated side. Scale bar =100 μm. The rectangle in the lower right corner of the figure for the resected side shows a magnified view of the area within the small rectangle.

Immunoreactivity for G-CSFR was much higher in the neural cells of the facial nerve nucleus on the treated (partially resected) side than in those on the non-treated side (Figure 6A). G-CSFR was also abundant in the peripheral nerve (buccal branch) on the treated (partially resected) side, whereas no G-CSFR expression was observed in the peripheral nerve on the non-neurectomy side (Figure 6B).

## 4 Discussion

The present study examined the effects of G-CSF on the morphological and functional recovery of severed facial nerves. The recovery of damaged nerves, evaluated by facial movements, CMAP amplitude measurements, ENoG values, and myelinated axon diameters, was faster in the severed-and-sutured groups than in the resection groups. Moreover, the recovery was faster in the G-CSF-treated group than in the control group, and in the group that received G-CSF immediately after surgical treatment than in the group that received G-CSF 2 weeks later. However, the group that received G-CSF 2 weeks later recovered faster than did the control group. G-CSFR was expressed in both the facial nerve nucleus and peripheral nerves on the nerve-resected side.

In the comparison between the different facial nerve damage groups, the severed-and-sutured groups showed significantly faster recovery for all the evaluated items than did the resection groups; this could be attributed to the fact that the regenerating buds of the axons can easily determine the extension direction. These findings suggest that, when axonal damage occurs, it is desirable to guide the destination of the regenerating axon by suturing (Grinsell and Keating, 2014; Faroni et al., 2015).

In the severed-and-sutured groups, the facial nerve function and the diameter of myelinated axons showed faster recovery in the G-CSF treated groups than in the control groups. These findings suggest that G-CSF promotes facial nerve regeneration. In addition, regarding the timing of G-CSF administration, recovery was faster in the group that received G-CSF immediately after the surgical treatment than in the group that received it 2 weeks later.

These findings suggest that G-CSF has a neuro-regenerative-promoting effect on injured facial nerves, and earlier G-CSF administration is desirable for faster functional recovery. Furthermore, the group that received G-CSF 2 weeks later recovered faster than the control group, which suggests that even a slight delay in the administration of G-CSF has a neuro-regenerative effect. This finding is consistent with previous reports, where Lee et al. revealed that delayed administration of G-CSF in the subacute phase of severe contusion spinal cord injury promoted spinal cord preservation and improved functional outcomes (Lee et al., 2012). Similarly, Koda et al. reported that delayed G-CSF treatment had a beneficial effect in an animal model of peripheral nerve injury-induced neuropathic pain (Koda et al., 2014).

Previous literature indicates that G-CSF has neuroprotective and nerve tissue repair properties (Solaroglu et al., 2006a; Aschauer-Wallner et al., 2021). Neuroprotective effects include anti-inflammatory and anti-apoptotic effects. G-CSF inhibits the expression of inflammatory cytokines (TNF-*α*, IL-1β) in inflammatory cells (Kadota et al., 2012), promotes upregulation of anti-apoptotic proteins, and prevents neuronal apoptosis, thereby exerting a direct neuroprotective effect on neurons (Solaroglu et al., 2006a; Nishio et al., 2007; Pitzer et al., 2010; Kadota et al., 2012). It also maintains the microenvironment by inhibiting oligodendrocyte death and protecting the myelin sheath (Kadota et al., 2012). With regard to neural tissue repair, G-CSF induces neurogenesis and angiogenesis (Wallner et al., 2015); moreover, it induces neural stem cell differentiation in neurons and promotes the recruitment of neural stem cells to nerve injury sites (Schneider et al., 2005a). In addition, G-CSF promotes neurite outgrowth (Pitzer et al., 2010), increases expression of angiogenic growth factors, contributes to angiogenesis and arteriogenesis (generation and recovery of large blood vessels such as small arteries), and promotes blood supply (Kawabe et al., 2011; Wallner et al., 2015). It has also been reported to mobilize bone marrow-derived cells and release cytokines and growth factors that assist in the regeneration of injured neural tissue (Koda et al., 2007). As described above, G-CSF has been reported to have the ability to protect and repair nerves, directly or indirectly.

According to Schneider et al., G-CSF and its receptors are expressed in neurons across many brain regions, with expression levels increasing after experimental stroke (Schneider et al., 2005b). The anti-apoptotic and protective activities of G-CSF are mediated by the G-CSFR in neurons. This was confirmed by the loss of G-CSF’s protective activity when antibodies against G-CSFR were used (Schneider et al., 2005a). In the present study, we measured G-CSFR to identify the sites of G-CSF activity within the facial nerve and confirmed that G-CSFR was expressed in facial nerve nuclei and peripheral nerves following partial facial nerve resection. This suggests that G-CSF exerts a direct effect on the entire population of motor neurons in the facial nerve.

As mentioned earlier, the present study demonstrated that early administration of G-CSF promoted early regeneration of the facial nerve. Schneider et al. reported that, in a rat model of acute stroke (middle cerebral artery occlusion), G-CSFR mRNA expression was induced as early as 6 h post-injury. Furthermore, although not neural, Hara et al. found that in mice with thigh muscle injury, G-CSFR expression peaked between 3 and 8 days post-injury (Hara et al., 2011). Considering that G-CSF suppresses neuronal apoptosis, provides neuroprotection, promotes neural tissue repair, and aids functional recovery, it is recommended that G-CSF be administered as early as possible after nerve injury. The mechanisms of G-CSF action outlined above likely contributed to the results of the present experiment, where early administration of G-CSF led to faster recovery of the facial nerve following injury.

Meanwhile, there is a possibility that there will be many cases where the start of treatment will be delayed because, in actual clinical practice, the time at which the severity of facial nerve paralysis can be determined is 7–10 days after onset; that is, after Wallerian degeneration. Therefore, the results of this experiment show that even if the administration of G-CSF is delayed, it still has the effect of promoting nerve regeneration, which is considered to be important in clinical practice.

In this study, the facial muscles showed a faster recovery from atrophy in the G-CSF group. There are two possible explanations for this observation. First, faster regeneration of the facial nerve may have induced faster regeneration of the facial muscles. Second, G-CSF may have had a direct effect on the muscle. The latter can be assumed because muscle and bone marrow cells are of mesodermal origin. Stratos et al. and Hara et al. reported that G-CSF promotes regeneration of injured skeletal muscles in the rat soleus muscle and mouse thigh muscle, respectively (Stratos et al., 2007; Hara et al., 2011).

This study had some limitations.

First, the G-CSF dose was based on information from studies using other nervous systems, and the optimal G-CSF dose for facial nerve regeneration in rats was not tested.

Second, synkinesis is a major sequela of severe facial paralysis. Even if regeneration can be promoted by various drugs, treatment satisfaction cannot be sufficiently achieved in severe cases unless this challenge is addressed. Therefore, further research is needed to address synkinesis and promote the regeneration of the facial nerve.

Third, in this experiment, a commercial drug delivery system was used, and as a result, the G-CSF concentration in the tissues was not measured over time. For the drug’s release profile, we relied on the reference data provided in the formulation instructions.

Fourth, while we referenced previous reports that suggest the inhibition of inflammatory factor expression as one of the mechanisms of action of G-CSF, we did not measure inflammatory factor levels in our experiments.

Fifth, although transmission electron microscopy provides detailed information on axonal and myelin sheath structures during facial nerve regeneration, we did not employ this technique in the current study because all samples were used for histological analysis of the effects of G-CSF.

Sixth, with the fluorescence staining method used in this study, we were unable to obtain sufficient resolution to confirm the expression of G-CSFR in the Schwann cells of the peripheral nerves. We hope to address this limitation in future research, because confirming the localization of G-CSFR expression in peripheral nerves can enhance our understanding of the role of G-CSF in nerve regeneration.

Seventh, because the main focus of this study was nerve regeneration, we did not perform a quantitative evaluation of muscle regeneration, although we recognize its importance. As reported in the literature, G-CSF can directly promote muscle regeneration, and we would like to evaluate and compare the direct effect of G-CSF on muscle recovery with its indirect effect via the promotion of nerve regeneration in future studies.

This study showed that G-CSF, both functionally and histomorphologically, has a neuro-regenerative-promoting effect on injured facial nerves, at least on nerves that have been reconstructed after amputation. The optimal dosage and timing of G-CSF administration in humans need to be investigated in the future.

G-CSF exerts its neuroregenerative effect through systemic administration. Therefore, when the use of G-CSF is considered in the clinical setting of facial nerve paralysis, there is no need for invasive procedures such as mastoidectomy or exposure of facial nerve branches, and additional administration of G-CSF can be easily performed when necessary. G-CSF has been used to treat hematological diseases in clinical settings. Accordingly, G-CSF for facial nerve palsy may be a therapeutic agent with the potential for early clinical application.

## Data Availability

The original contributions presented in the study are included in the article/supplementary material, further inquiries can be directed to the corresponding author.

## Footnotes

^1^ https://imagej.nih.gov/ij/

^2^ www.graphpad.com
